# Threonine Affects Intestinal Function, Protein Synthesis and Gene Expression of TOR in Jian Carp (*Cyprinus carpio* var. Jian)

**DOI:** 10.1371/journal.pone.0069974

**Published:** 2013-07-26

**Authors:** Lin Feng, Yan Peng, Pei Wu, Kai Hu, Wei-Dan Jiang, Yang Liu, Jun Jiang, Shu-Hong Li, Xiao-Qiu Zhou

**Affiliations:** 1 Animal Nutrition Institute, Sichuan Agricultural University, Chengdu, Sichuan, China; 2 Fish Nutrition and Safety Production University Key Laboratory of Sichuan Province, Sichuan Agricultural University, Chengdu, Sichuan, China; 3 Key Laboratory for Animal Disease-Resistance Nutrition of China Ministry of Education, Sichuan Agricultural University, Chengdu, Sichuan, China; The University of Plymouth, United Kingdom

## Abstract

This study aimed to investigate the effects of threonine (Thr) on the digestive and absorptive ability, proliferation and differentiation of enterocytes, and gene expression of juvenile Jian carp (*Cyprinus carpio* var. Jian). First, seven isonitrogenous diets containing graded levels of Thr (7.4–25.2 g/kg diet) were fed to the fishes for 60 days. Second, enterocyte proliferation and differentiation were assayed by culturing enterocytes with graded levels of Thr (0–275 mg/l) *in vitro*. Finally, enterocytes were cultured with 0 and 205 mg/l Thr to determine protein synthesis. The percent weight gain (PWG), specific growth rate, feed intake, feed efficiency, protein retention value, activities of trypsin, lipase and amylase, weights and protein contents of hepatopancreas and intestine, folds heights, activities of alkaline phosphatase (AKP), γ- glutamyl transpeptidase and Na^+^/K^+^-ATPase in all intestinal segments, glutamate-oxaloacetate transaminase (GOT) and glutamate-pyruvate transaminase (GPT) activities in hepatopancreas, and *4E-BP2* gene expression in muscle, hepatopancreas and intestinal segments were significantly enhanced by Thr (*p*<0.05). However, the plasma ammonia concentration and *TOR* gene expression decreased (*p*<0.05). *In vitro*, Thr supplement significantly increased cell numbers, protein content, the activities of GOT, GPT, AKP and Na^+^/K^+^-ATPase, and protein synthesis rate of enterocytes, and decreased LDH activity and ammonia content in cell medium (*p*<0.05). In conclusion, Thr improved growth, digestive and absorptive capacity, enterocyte proliferation and differentiation, and protein synthesis and regulated *TOR* and *4E-BP2* gene expression in juvenile Jian carp. The dietary Thr requirement of juvenile Jian carp was 16.25 g/kg diet (51.3 g/kg protein) based on quadratic regression analysis of PWG.

## Introduction

Fish growth is related to the capacity of fish digestive system to break down and assimilate nutrients, which partly depends on digestive and brush border enzymes activities [1]. In fish, trypsin, lipase and amylase are major digestive enzymes that respond to degrading dietary proteins, lipids and carbohydrates, respectively [2]. Intestinal brush border enzymes, such as alkaline phosphatase (AKP) [3], γ-glutamyl transpeptidase (γ-GT) [4] and Na^+^/K^+^-ATPase [5], are involved in nutrient absorption in fish. In stomachless fishes, the intestine plays a central role in digesting and absorbing nutrients [6]. The fish intestinal epithelium, a site for nutrients uptake, is expanded by folding. Gut folds is regarded as a sign of absorption ability in fish [7]. To date, the understanding of the development of digestive organs and the activity of digestive enzymes in fish is gradually improving [1]. However, there are few studies concerning the factors that influence the digestive and absorptive functions of fish.

The digestive enzyme activity of fish larvae can be affected by dietary composition [8]. Threonine (Thr) is an essential amino acid (EAA) for fish [9]. Additionally, Thr is assumed to be one of the most common limiting amino acids in some practical diets for fish, which have high levels of plant proteins instead of fishmeal [10]. Accordingly, Thr is a vital dietary component for fish. However, there is no information about the relationship between Thr and digestive-absorptive enzymes in fish. Studies have shown that Thr participated in amino acid composition of intestinal AKP in calves [11], and was necessary for amylase synthesis in pigeon pancreas [12]. Furthermore, the digestive and absorptive functions of fish are largely dependent on the growth and development of digestive organs [13]. Thr-deficient diet decreased the gut weight of rats [14] and the midjejunum villus heights of neonatal piglets [15]. In piglets, Thr is the amino acid that is used to the greatest extent by the portal-drained viscera (including the intestines and pancreas), and 60–80% of dietary Thr is extracted by the portal-drained viscera on the first pass [16]. Meanwhile, intestinal mucins are particularly enriched in Thr (up to 30% of the amino acid composition) [14]. Thus, Thr may play an important role in the growth and development of digestive organs in fish, which has not yet been investigated. In fish digestive system, the intestinal epithelium represents a cell renewal system, in which cells proliferate rapidly and have short half-lives [17]. Enterocytes are the main intestinal columnar epithelium cells and key cells for nutrient digestion and absorption [17]. Therefore, the proliferation and differentiation of enterocytes are very important for the growth, development and function of intestine. Johnson [18] suggested that enterocytes growth alteration factors are of paramount importance to digestive and absorptive abilities. In weanling pigs, dietary Thr deficiency reduced the acidomucin goblet cell counts in ileum and duodenum [19]. Nevertheless, no studies have been carried out in fish to investigate the effect of Thr on the proliferation and differentiation of enterocytes, which is worthy of investigation.

The developmental growth of fish tissues and organs rely on protein synthesis [20]. In animals, a large proportion of intestinal amino acids are used for constitutive gut growth through participating in protein synthesis [21]. Piglet intestine could utilize dietary Thr for intestinal protein synthesis [16]. A low Thr diet decreased the total protein content in jejunum of rat [14] and the liver protein content of piglets [22]. Recent studies from our laboratory indicated that the hepatopancreas and intestinal protein contents of Jian carp were improved by dietary lysine [23] and arginine [24]. However, no information has been reported about the effect of Thr on digestive organ protein synthesis in fish. Protein synthesis in mammals is limited by the initiation step of translation, which is regulated by the target of rapamycin (TOR) signaling pathway [25]. TOR protein plays a central role in the TOR signaling pathway [25], and eIF4-binding protein (4E-BP) can be hyperphosphorylated by the activated TOR protein resulting in translation initiation [26]. Recently, the *TOR* (GenBank Number FJ899680) and *4E-BP2* (Genbank Number HQ010440) genes of Jian carp were firstly cloned in our laboratory. Our studies also showed that muscle protein content was positively related to the relative expression of *TOR* gene in fish muscle (J. Jiang, unpublished). Mammal TOR protein is an evolutionarily conserved Ser/Thr kinase, and Thr is essential for its activity [27]. Meanwhile, the activity of mammal 4E-BP1 also depended on several Thr residues [28]. Studies from our laboratory showed that *TOR* gene expression of Jian carp was regulated by arginine *in vivo* [24] and by glutamine *in vitro* [29]. These data show that there may be a close relationship between Thr and *TOR*/*4E-BP* in fish digestive organs, which has not yet been characterized.

The present study aimed to elucidate the effects of Thr on fish digestion and absorption and the possible mechanisms underlying these effects. Thus, we conducted three experiments *in vivo* and *in vitro* to investigate the effects of Thr on the digestive and absorptive capacity, enterocyte proliferation and differentiation, and *TOR* and *4E-BP2* gene expression in juvenile Jian carp as a mean of establishing a foundation of the effect of Thr on fish digestive and absorptive functions.

## Materials and Methods

### Ethics statement

All experimental procedures were approved by the Animal Care Advisory Committee of Sichuan Agricultural University.

### Chemicals

Casein, gelatin and L-crystalline amino acids were purchased from Hulunbeier Sanyuan Milk Co., Ltd. (Inner Mongolia, China), Rousselot Gelatin Co., Ltd. (Guangdong, China) and Donboo Amino Acid Co. Ltd. (Jiangsu, China), respectively. Microencapsulated Thr was purchased from Chongqing Medicines & Health Products Imp. & Exp. Co., Ltd. (Chongqing, China). L-Thr, insulin, collagenase, dispase, collagen, benzyl penicillin, streptomycin sulfate, transferrin, D-sorbitol, HEPES, Triton X-100, dimethyl sulfoxide (DMSO), 3-(4, 5-dimethylthiazol-2-yl)-2, 5-diphenyltetrazolium bromide (MTT) and [^3^H]phenylalanine were purchased from Sigma (St. Louis, MO, USA). Dulbecco's Modified Eagle Medium (DMEM) and fetal bovine serum (FBS) were obtained from Hyclone (Logan, UT, USA). Thr-free DMEM was ordered from Beijing Tsing Skywing Bio. Tech. Co., Ltd (Beijing, China).

### *In vivo* experiment

#### Diets and fish

Dietary protein was supplied by casein, gelatin and L-crystalline amino acids with a minimum quantity of Thr (Table S1). The dietary amino acid profile was similar to that of whole egg protein containing 320 g crude protein/kg with the exception of Thr by supplementing L-crystalline amino acids [30]. Microencapsulated Thr (containing 498.2 g Thr/kg) was added to the basal diet to provide seven diets with graded levels of Thr (7.0 (Thr-deficient group), 9.5, 12.5, 15.5, 18.5, 21.5 and 24.5 g/kg diet). The seven diets were maintained isonitrogenous by varying the amounts of aspartic acid, glutamic acid and alanine. Pyridoxine, inositol, riboflavin and pantothenic acid were supplemented to meet the requirements of juvenile Jian carp according to our laboratory's studies [31], [32], [33], [34]. Other nutrients met the requirements of common carp (*Cyprinus carpio*) according to NRC [35]. The analyzed values of Thr in these diets were 7.4, 9.1, 12.2, 15.7, 18.6, 22.3 and 25.2 g/kg diet according to high-performance liquid chromatography (L-8800, Hitachi, Tokyo, Japan) analysis [36]. The pH of each diet was adjusted to 7.0 by adding NaOH, and pellets were then produced and stored at −20°C until use [37].

Fish management was followed the Guidelines for the Care and Use of Laboratory Animals of Sichuan Agricultural University as described by Wu et al. [38]. Juvenile Jian carp were obtained from Ya'an fisheries (Sichuan, China) and acclimatized for 4 weeks. A total of 1050 fish (with an average initial weight of 13.60±0.03 g) were randomly assigned to 21 experimental aquaria (90 L×30 W×40 H cm) each of which was connected to a closed recirculating water system with continuous aeration. Each of the seven diets was fed to three replicates of fish six times daily to apparent satiation for 60 days, and uneaten feed was removed by siphoning after each meal. The dissolved oxygen was higher than 5 mg/l. The water temperature was 25±1°C. Other water quality characteristics were monitored and maintained at acceptable levels. A 14∶10 h light-dark photoperiod was maintained.

#### Body composition and enzyme activities

The fish in each aquarium were weighed at the beginning and end of the feeding trial. The sampling procedures followed the method described by Jiang et al. [39]. Before sampling, fish were anesthetized in a benzocaine bath (50 mg/l) after being fasted for 12 h. Thirty fish from the same population before the experiment and five fish from each aquarium at the end of feeding trial were selected for determination of initial and final carcass proximate composition respectively. The proximate compositions of feed and fish carcass were analyzed according to the method of AOAC [40]. Another 15 fish from each aquarium were selected 12 h after the last feeding. Muscle, hepatopancreas and intestine were quickly removed, weighed and frozen in liquid nitrogen, then stored at −70°C for analysis. Another 5 fish were collected from each aquarium and intestines were sampled to measure the intestinal folds height according to Lin and Zhou [41]. Six hours after the last feeding, blood was drawn from the caudal vein of 5 fish from each aquarium using heparinized syringes. Plasma was recovered after centrifugation at 4000 *g* for 15 min at 4°C and used to assay plasma ammonia content as described by Bergmeyer and Beutler [42].

Intestine, hepatopancreas and muscle samples were homogenized in 10 volumes (w/v) of ice-cold physiological saline and centrifuged at 6000 *g* for 20 min at 4°C and the supernatant was used to assay enzyme activities. Trypsin activity was determined according to Hummel [43], and lipase and amylase activities were assayed according to Furné et al. [44]. Na^+^/K^+^-ATPase, AKP and γ-glutamyl transpeptidase (γ-GT) activities were determined using the procedure of Mccormick [45], Bessey et al. [46] and Bauermeister et al. [47], respectively. The protein content was determined as described by Bradford [48]. The activities of GOT and GPT were detected according to Bergmeyer and Bernt [49], [50], respectively.

#### Gene expression

Total RNA was isolated from the muscle, hepatopancreas and all intestinal segments by using RNAiso plus Kit (TaKaRa, Dalian, China). The quality and quantity of RNA was assessed by spectrophotometry at 260 and 280 nm and electrophoresis on 1% agarose gels. cDNA was synthesized with 2 µl of total RNA by using the PrimeScript™ RT reagent Kit (TaKaRa, Dalian, China). Real-time PCR was performed with a chromo 4™ continuous fluorescence detector (Bio-Rad, Laboratories, Inc.) according to standard protocols with the primers and thermocycling conditions indicated in Table S2. The cDNA (2 µl) was reacted with forward and reverse primers, SYBR Premix Ex Taq™ II (2×) (7.5 µl) (TaKaRa, Dalian, China) and RNase free dH_2_O in a total reaction volume of 15 µl. The melting curve analysis was performed over a range of 50–95°C to verify that a single PCR product was generated. All samples were run in parallel with the housekeeping gene (β-actin, GenBank Number M24113) to normalize cDNA loading. The expression results were analyzed using the 2^−ΔΔCt^ method after verification that the primers were amplified with an efficiency of approximately 100% [51]. The amplification efficiencies of the target and housekeeping genes were calculated according to the specific gene standard curves generated from 10-fold serial dilutions.

### *In vitro* experiments

#### Cell isolation, culture and stimulations

The cell isolation and culture procedures were based on a method established in our laboratory as described by Chen et al. [52]. Healthy Jian carp (50–60 g) were purchased from a local hatchery. After starvation for 24 h, the fish were killed and the intestines were rapidly removed, opened and rinsed with Hanks balanced salt solution containing 100 µg streptomycin sulfate/ml and 100 IU benzyl penicillin/ml. Enterocytes were isolated by collagenase and dispase dissociation, and then suspended in DMEM and washed with the same medium 5 times. Isolated enterocytes were plated in DMEM supplemented with 5% FBS, 0.02 mg transferrin/ml, 0.01 mg insulin/ml, 100 IU benzyl penicillin/ml and 100 µg streptomycin sulfate/ml, and then cultured at 26±0.5°C in 24-well culture plates (Falcon) for 72 h.

For analyzing the proliferation and differentiation of enterocytes, after 72 h, cells were incubated in six new media containing graded levels of Thr (0, 135, 170, 205, 240 or 275 mg/l) (n = 4) for 96 h at 26±0.5°C. After the incubation, cell lysates and media were collected as described by Jiang et al. [53]. Cell lysates were used for analysis of protein content and activities of AKP, Na^+^/K^+^-ATPase, GOT and GTP. The analysis methods were the same as described above. Cell media were used for analysis of ammonia content and LDH activity. LDH activity was measured by the method of Wróblewski and Ladue [54].

For protein synthesis analysis, cells were incubated with new media containing 0 and 205 mg/l Thr (n = 8) at 26±0.5°C for 4 h according to the preliminary experiment (data not shown). The Thr concentration was determined according to the results of the experiment about enterocyte proliferation and optimal for cell proliferation.

#### Cell proliferation assay

Cell proliferation was measured by MTT assay as described by Daly et al. [55]. In brief, the medium was removed and replaced by 500 µl MTT working solution at the end of the incubation. After incubation for 4 h, the MTT working solution was removed, and then 500 µl DMSO was added to dissolve the formazan precipitates. The amount of formazan was determined by measuring optical density (OD) at 595 nm.

#### Protein synthesis analysis

The protein synthesis rate was determined as the method described by Higashiguchi et al. [56]. In brief, 50 µL 100 mmol/l Phe (containing 3.70 MBq/ml [^3^H]Phe) was added to each cell culture resulting in a total of 1.11×10^7^ disintegrations per minute (DPM). The incubation was carried out at 26°C in a shaking water bath for 4 h and stopped by 1 ml 20% perchloric acid. After centrifugation at 2000 *g* for 5 min at 4°C, the precipitated pellet was washed three times with 0.5 mol/l perchloric acid and centrifuged again. Finally, the pellet was solubilized in 0.3 mol/l NaOH and left to stand for 12 h, and then the sample was mixed with scintillation fluid (OptiPhase HiSafe 3, PerkinElmer, USA) for measuring radioactivity by 2000CA Tri-Carb liquid scintillation analyzer (PerkinElmer, USA). The enterocyte protein synthesis rate was expressed in DPM per µg of protein.

### Calculations and statistical analysis

Data on the initial body weight, final body weight, feed intake (FI), proximate composition of feed and carcass, morphological measurements and protein concentrations of hepatopancreas and intestine were used to calculate the following parameters:
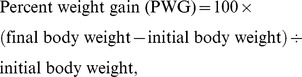
(1)

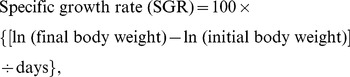
(2)

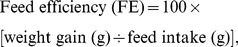
(3)

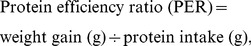
(4)

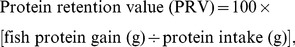
(5)

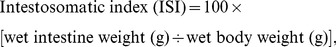
(6)

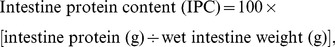
(7)

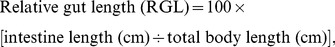
(8)

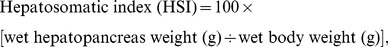
(9)

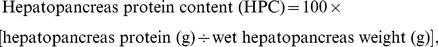
(10)


Results were presented as means ± SD. The protein synthesis rate of enterocytes was subjected to Student's *t* test. Other data were subjected to one-way analysis of variance followed by Duncan's method to determine significant differences among treatments at the level of *p*<0.05 through SPSS 13.0 (SPSS Inc., Chicago, IL, USA). Second-degree polynomial regression analysis was employed to estimate the Thr requirement of juvenile Jian carp [57].

## Results

### *In vivo* analysis

#### Growth performance

**Figure 1:**
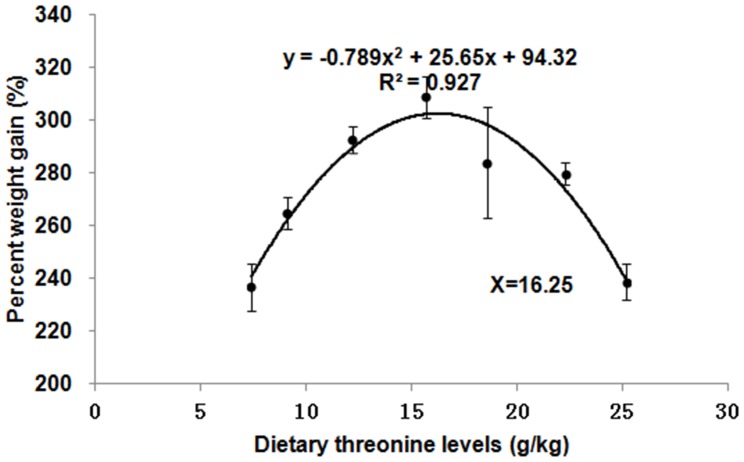
Second-degree polynomial regression analysis of percent weight gain (PWG) for juvenile Jian carp fed with graded levels of Thr. Each point represents the mean of three replicates with fifty fish per replicate.

Growth performance, final body composition and the survival of juvenile Jian carp that were fed diets containing graded levels of Thr are given in Table 1. No mortality was observed during the experiment. PWG, SGR, FI, fish final body protein and lipid contents, and PRV significantly improved with increased dietary Thr level up to 15.7 g/kg diet, and then decreased (*p*<0.001); whereas, the moisture and ash contents showed the opposite pattern with the lowest in fish fed with 15.7 g Thr/kg diet (moisture, *p*<0.001; ash, *p* = 0.07). The FE and PER were also improved by Thr and the highest in the group fed with 22.3 g Thr/kg diet (FE, *p* = 0.045; PER, *p* = 0.072). Furthermore, the PWG, SGR, FI, FE, PER, final body protein, lipid, ash and moisture content, and PRV showed quadratic response to dietary Thr levels (Table 1). The dietary Thr requirement of juvenile Jian carp was estimated to be 16.25 g/kg diet (51.3 g/kg protein) by quadratic regression analysis based on PWG (Figure 1).

**Table 1:** Growth performance, final body composition and survival of juvenile Jian carp fed diets containing graded levels of Thr.

Thr (g/kg)	7.4	9.1	12.2	15.7	18.6	22.3	25.2	*p*-value
IBW (g)^1^	13.6^a^±0.0	13.6^a^±0.0	13.6^a^±0.0	13.6^a^±0.0	13.6^a^±0.0	13.6^a^±0.0	13.6^a^±0.1	= 0.917
FBW (g)^1^	45.9^a^±1.0	49.7^b^±0.7	53.4^cd^±0.5	55.6^d^±0.9	52.2^bc^±2.3	51.7^bc^±0.5	46.1^a^±0.8	<0.001
PWG^1^	237±9^a^	265±6^b^	293±5^cd^	309±8^d^	284±21^bc^	280±4^bc^	239±7^a^	<0.001
SGR^1^	2.02^a^±0.04	2.16^b^±0.02	2.28^cd^±0.02	2.35^d^±0.02	2.24^bc^±0.07	2.22^bc^±0.02	2.03^a^±0.10	<0.001
FI (g)^1^	51.5^a^±0.3	55.3^b^±0.3	59.6^d^±0.4	62.4^e^±0.4	57.3^c^±0.6	55.6^b^±0.1	51.4^a^±0.9	<0.001
FE (%)^1^	62.6^a^±2.1	65.3^abc^±2.0	66.8^abc^±1.3	67.4^bc^±1.3	67.4^bc^±4.8	68.5^c^±0.9	63.2^ab^±0.8	= 0.045
PER^1^	1.98^a^±0.08	2.06^ab^±0.05	2.11^ab^±0.03	2.13^ab^±0.05	2.13^ab^±0.16	2.16^b^±0.03	2.00^a^±0.06	= 0.072
Moisture (g/kg)^2^	743^b^±8	737^ab^±9	735^ab^±8	727^a^±7	738^ab^±8	743^b^±10	768^c^±7	<0.001
CP (g/kg)^2^	151^b^±1	152^b^±2	153^bc^±2	155^c^±2	152^b^±1	151^b^±1	144^a^±1	<0.001
Lipid (g/kg)^2^	89.2^ab^±3.8	92.3^b^±6.4	109^c^±4	113^c^±6	95.8^b^±4.6	91.2^ab^±4.8	85.0^a^±6.3	<0.001
Ash (g/kg)^2^	25.7^b^±1.2	25.6^b^±1.5	24.1^ab^±0.8	23.5^a^±0.9	24.6^ab^±1.2	24.3^ab^±1.2	24.6^ab^±1.3	= 0.07
PRV (%)^2^	30.0^b^±0.4	31.5^c^±0.6	32.5^d^±0.7	33.3^e^±0.5	32.3^d^±0.2	32.8^de^±0.3	28.3^a^±0.3	<0.001
Survival (%)^2^	100^a^±0	100^a^±0	100^a^±0	100^a^±0	100^a^±0	100^a^±0	100^a^±0	—
Regressions								
Y_PWG_ = −0.789x^2^+25.65x+94.32					R^2^ = 0.927		*p*<0.01	
Y_SGR_ = 1.3705+0.1177x−0.0036x^2^					R^2^ = 0.927		*p*<0.01	
Y_FI_ = 31.33+3.665x−0.115x^2^					R^2^ = 0.889		*p*<0.05	
Y_FE_ = 51.50+1.970x−0.058x^2^					R^2^ = 0.790		*p*<0.05	
Y_PER_ = 1.623+0.062x−0.002x^2^					R^2^ = 0.811		*p*<0.05	
Y_CP_ = 137.81+2.307x−0.080x^2^					R^2^ = 0.908		*p*<0.01	
Y_Lipid_ = 43.8+8.135x−0.264x^2^					R^2^ = 0.743		*p* = 0.066	
Y_Ash_ = 29.243−0.592x+0.017x^2^					R^2^ = 0.725		*p* = 0.076	
Y_Moisture_ = 791.79−8.691x+0.303x^2^					R^2^ = 0.922		*p*<0.01	
Y_PRV_ = 21.382+1.523x−0.048x^2^					R^2^ = 0.820		*p*<0.05	

1 Values are means ± SD, means of three replicates with fifty fish per replicate. Mean values with the different superscripts in the same row are significantly different (*p*<0.05).

2 Values are means ± SD, n = 5. Mean values with the different superscripts in the same row are significantly different (*p*<0.05). IBW, Initial body weight; FBW, final body weight; PWG, percent weight gain; SGR, specific growth rate; FI, feed intake; FE, feed efficiency; PER, protein efficiency ratio; CP, crude protein; PRV, protein retention value.

#### Hepatopancreas and intestine weight

As shown in Table 2, hepatopancreas weight (HW), intestine weight (IW), intestinal length (IL), RGL, HSI and ISI were significantly enhanced as the Thr level increased up to 15.7 g/kg diet, and then decreased (*p*<0.001). The HPC, IPC and folds heights in proximal intestine (PI) and mid intestine (MI) were increased in response to the increase of Thr level and the maximum in group fed with 15.7 g Thr/kg diet (*p*<0.001). The folds heights in the distal intestine (DI) significantly increased as the Thr level increased to 12.2 g/kg diet (*p*<0.001), but no significant differences occurred from 12.2 to 22.3 g Thr/kg diet. Regression analysis showed that the HW, IW, IL, RGL, HSI, HPC, IPC, and the folds heights in PI, MI and DI quadratically responded to dietary Thr levels (Table 2).

**Table 2:** Growth and development of hepatopancreas and intestine, and intestinal folds height of juvenile Jian carp fed diets containing graded levels of Thr.

Thr (g/kg)	7.4	9.1	12.2	15.7	18.6	22.3	25.2	*p*-value
Hepatopancreas								
HW (g)^1^	1.24^a^±0.10	1.52^b^±0.11	1.83^c^±0.15	2.02^d^±0.20	1.86^c^±0.19	1.76^c^±0.11	1.47^b^±0.12	<0.001
HSI (%)^1^	27.0^a^±1.5	30.7^b^±2.6	34.3^de^±1.6	36.4^e^±2.5	35.4^de^±1.9	33.8^cd^±3.3	32.1^bc^±2.2	<0.001
HPC(%)^2^	61.2^a^±1.1	68.6^bc^±1.5	74.2^d^±1.4	77.9^e^±3.7	71.9^cd^±3.1	68.5^bc^±2.6	67.6^b^±3.6	<0.001
Intestine								
IL (cm)^1^	16.7^a^±0.9	19.3^bc^±0.7	21.0^d^±1.3	22.2^e^±1.0	20.5^d^±1.4	20.2^cd^±1.1	18.9^b^±1.1	<0.001
RGL(%)^1^	137^a^±7	152^bc^±8	162^de^±11	170^e^±6	163^de^±8	156^cd^±9	146^b^±8	<0.001
IW (g)^1^	0.80^a^±0.10	0.88^bc^±0.08	1.06^e^±0.07	1.20^f^±0.07	0.97^d^±0.09	0.94^cd^±0.07	0.81^ab^±0.09	<0.001
ISI(%)^1^	17.1^a^±1.6	17.9^ab^±0.6	19.6^c^±0.9	21.6^d^±1.6	18.7^bc^±0.6	18.4^b^±1.3	17.8^ab^±1.5	<0.001
IPC(%)^2^	61.2^a^±1.1	65.5^bc^±1.4	78.5^e^±2.0	85.9^f^±4.0	70.0^d^±1.9	69.5^cd^±3.5	64.3^ab^±5.2	<0.001
Intestinal folds height (µm)^2^								
PI	522^a^±18	680^b^±29	854^de^±25	874^e^±30	822^cd^±34	793^c^±29	550^a^±27	<0.001
MI	367^a^±10	447^b^±15	491^c^±19	532^d^±18	480^c^±15	470^c^±21	386^a^±15	<0.001
DI	317^a^±20	328^a^±24	434^b^±20	438^b^±19	426^b^±21	421^b^±22	323^a^±24	<0.001
Regressions								
Y_HW_ = −0.236+0.261x−0.008x^2^					R^2^ = 0.966		*p*<0.01	
Y_IW_ = 0.086+0.126x−0.004x^2^					R^2^ = 0.806		*p*<0.05	
Y_IL_ = 8.132+1.595x−0.047x^2^					R^2^ = 0.863		*p*<0.05	
Y_RGL_ = 77.756+10.794x−0.324x^2^					R^2^ = 0.945		*p*<0.01	
Y_HSI_ = 10.759+2.889x−0.082x^2^					R^2^ = 0.961		*p*<0.01	
Y_HPC_ = 36.261+4.696x−0.141x^2^					R^2^ = 0.788		*p*<0.05	
Y_IPC_ = 20.655+7.185x−0.221x^2^					R^2^ = 0.702		*p* = 0.089	
Y_Intestinal folds height (PI)_ = −255.78+139.78x−4.2565x^2^					R^2^ = 0.941		*p*<0.01	
Y_Intestinal folds height (MI)_ = 65.013+55.472x−1.696x^2^					R^2^ = 0.913		*p*<0.01	
Y_Intestinal folds height (DI)_ = −5.378+53.858x−1.604x^2^					R^2^ = 0.896		*p*<0.05	

1 Values are means ± SD, n = 15. Mean values with the different superscripts in the same row are significantly different (*p*<0.05).

2 Values are means ± SD, n = 5. Mean values with the different superscripts in the same row are significantly different (*p*<0.05). HW, hepatopancreas weight; HSI, hepatosomatic index; HPC, hepatopancreas protein content; IW, intestine weight; IL, intestine length; RGL, relative gut length; ISI, intestosomatic index; IPC, intestine protein content; PI, proximal intestine; MI, mid intestine; DI, distal intestine.

#### Plasma ammonia concentration and enzymes activities

The plasma ammonia concentration (PAC), and GOT and GPT activities in hepatopancreas also significantly responded to dietary Thr (Table 3). GOT activity in hepatopancreas significantly increased in response to increased Thr levels up to 12.2 g/kg diet (*p*<0.001). The activity of GPT in hepatopancreas showed a similar pattern as GOT and was the highest in the group containing 15.7 g Thr/kg diet (*p*<0.001). However, the PAC was significantly decreased with the increase of Thr levels, and the lowest in fish fed with 15.7 g Thr/kg diet (*p*<0.001). Regression analysis showed that GOT and GPT activities in hepatopancreas, and PAC quadratically responded to dietary Thr levels (Table 3).

**Table 3:** Glutamate-oxaloacetate transaminase (GOT) and glutamate-pyruvate transaminase (GPT) activities in hepatopancreas and plasma ammonia concentration (PAC) of juvenile Jian carp fed diets containing graded levels of Thr.

Thr (g/kg)	7.4	9.1	12.2	15.7	18.6	22.3	25.2	*p*-value
GOT (U/g tissue)	2525^a^±96	3039^b^±96	4237^e^±122	4366^e^±159	3767^d^±140	3510^c^±48	3381^c^±122	<0.001
GPT (U/g tissue)	957^a^±26	976^ab^±34	1014^bc^±40	1062^c^±40	1053^c^±43	1024^bc^±48	957^a^±43	<0.001
PAC (µmol/l)	2129^d^±127	1576^c^±64	1341^b^±64	1000^a^±40	1529^c^±81	1646^c^±83	2446^e^±174	<0.001
Regressions								
Y_GOT_ = −578.64+558.49x−16.327x^2^					R^2^ = 0.787		*p*<0.05	
Y_GPT_ = 703.6+42.11x−1.261x^2^					R^2^ = 0.948		*p*<0.01	
Y_PAC_ = 4392−418.4x+13.499x^2^					R^2^ = 0.908		*p*<0.01	

Values are means ± SD, n = 5. Mean values with the different superscripts in the same row are significantly different (*p*<0.05).

As presented in Table 4, the activities of trypsin, lipase and amylase in intestine and hepatopancreas were increased significantly with increased dietary Thr levels up to 15.7 g/kg diet and decreased significantly with further increase of Thr levels (*p*<0.001). The activities of trypsin, lipase and amylase in intestine and hepatopancreas responded quadractically to increased dietary Thr levels (Table 4). The brush border enzymes activities of juvenile Jian carp fed diets containing graded levels of Thr are shown in Table 5. The Na^+^/K^+^-ATPase activities in PI and MI were significantly enhanced as Thr levels increased to 15.7 g/kg diet and then decreased significantly (*p*<0.001). The activities of Na^+^/K^+^-ATPase in DI, and AKP in PI, MI and DI significantly increased with increment of Thr levels up to 12.2 g/kg diet (*p*<0.001). The maximum activities of γ-GT in all intestinal segments were recorded in the group with 15.7 g Thr/kg diet (*p*<0.001). These enzymes showed quadratic responses to the increased dietary Thr levels (Table 5).

**Table 4:** The activities of trypsin, lipase and amylase in hepatopancreas and intestine of juvenile Jian carp fed diets containing graded levels of Thr.

Thr (g/kg)	7.4	9.1	12.2	15.7	18.6	22.3	25.2	*p*-value
Trypsin (U/g tissue)								
Hepatopancreas	2.41^a^±0.09	2.68^b^±0.11	2.72^bc^±0.09	2.85^c^±0.08	2.63^b^±0.14	2.44^a^±0.13	2.41^a^±0.16	<0.001
Intestine	2.42^a^±0.14	2.61^b^±0.14	2.93^c^±0.14	3.15^d^±0.12	2.91^c^±0.10	2.70^b^±0.07	2.57^ab^±0.13	<0.001
Lipase (U/g tissue)								
Hepatopancreas	496^a^±25	594^b^±19	642^b^±44	691^c^±29	638^b^±40	607^b^±37	540^a^±49	<0.001
Intestine	261^a^±10	323^bc^±20	372^d^±19	403^e^±24	350^cd^±29	319^b^±25	283^a^±19	<0.001
Amylase (U/g tissue)								
Hepatopancreas	1664^a^±65	1711^a^±47	1828^bc^±35	1877^c^±31	1791^b^±26	1718^a^±55	1708^a^±62	<0.001
Intestine	1067^a^±42	1149^bc^±22	1213^d^±17	1247^e^±9	1156^c^±23	1174^c^±17	1121^b^±26	<0.001
Regressions								
Y_Intestinal trypsin_ = 1.075+0.2367x−0.007x^2^					R^2^ = 0.895		*p*<0.05	
Y_Intestinal lipase_ = 16.303+45.467x−1.406x^2^					R^2^ = 0.879		*p*<0.05	
Y_Intestinal amylase_ = 804.06+49.988x−1.504x^2^					R^2^ = 0.730		*p* = 0.073	
Y_Hepatopancreas trypsin_ = 1.767+0.130x−0.004x^2^					R^2^ = 0.747		*p* = 0.064	
Y_Hepatopancreas lipase_ = 158.99+62.479x−1.894x^2^					R^2^ = 0.915		*p*<0.01	
Y_Hepatopancreas amylase_ = 1282.8+67.751x−2.073x^2^					R^2^ = 0.797		*p*<0.05	

Values are means ± SD, n = 5. Mean values with the different superscripts in the same row are significantly different (*p*<0.05).

**Table 5:** The brush border enzymes activities of juvenile Jian carp fed diets containing graded levels of Thr.

Thr (g/kg)	7.4	9.1	12.2	15.7	18.6	22.3	25.2	*p*-value
Na^+^/K^+^-ATPase (µmol phosphorus released/g tissue/hour)								
PI	133^a^±13	182^b^±11	225^c^±13	246^d^±13	225^c^±11	216^c^±14	177^b^±8	<0.001
MI	147^a^±14	196^b^±27	260^cd^±14	273^d^±26	252^cd^±16	233^c^±17	192^b^±20	<0.001
DI	92^a^±9	120^b^±14	140^c^±9	154^c^±11	145^c^±7	112^b^±11	98^a^±11	<0.001
AKP (mmol of nitrophenol released/g tissue/hour)								
PI	14.9^a^±0.7	16.7^b^±1.2	23.6^d^±0.6	24.8^d^±1.0	21.6^c^±1.8	16.8^b^±1.4	15.0^a^±0.7	<0.001
MI	10.3^a^±0.9	12.8^bc^±0.7	14.4^d^±1.0	14.8^d^±1.1	13.8^cd^±1.1	12.0^b^±0.7	10.5^a^±0.4	<0.001
DI	2.89^a^±0.17	3.26^abc^±0.17	3.88^d^±0.35	3.88^d^±0.41	3.63^cd^±0.46	3.45^bcd^±0.26	3.14^ab^±0.26	<0.001
γ-GT (mmol of 5-amino-2-nitrobenzoate released/g tissue/min)								
PI	29.8^a^±1.8	36.5^b^±2.4	44.7^cd^±2.5	47.7^d^±1.8	43.9^c^±4.4	35.3^b^±2.4	28.4^a^±1.6	<0.001
MI	50.0^a^±3.9	57.8^b^±4.5	67.2^cd^±2.7	71.9^d^±2.6	65.9^c^±3.4	58.4^b^±5.2	53.9^ab^±3.5	<0.001
DI	60.3^a^±2.1	67.5^b^±2.7	79.5^cd^±6.3	82.8^d^±7.1	74.8^c^±4.0	67.7^b^±3.9	60.9^a^±3.6	<0.001
Regressions								
Y_Na_^+^_/K_^+^_-ATPase (PI)_ = −68.602+36.399x−1.063x^2^					R^2^ = 0.945		*p*<0.01	
Y_Na_^+^_/K_^+^_-ATPase (MI)_ = −92.817+43.051x−1.270x^2^					R^2^ = 0.943		*p*<0.01	
Y_Na_^+^_/K_^+^_-ATPase (DI)_ = −32.245+22.655x−0.704x^2^					R^2^ = 0.936		*p*<0.01	
Y_γ-GT (PI)_ = −9.741+7.111x−0.224x^2^					R^2^ = 0.971		*p*<0.01	
Y_γ-GT (MI)_ = 8.814+7.431x−0.228x^2^					R^2^ = 0.917		*p*<0.01	
Y_γ-GT (DI)_ = 15.314+8.121x−0.254x^2^					R^2^ = 0.911		*p*<0.01	
Y_AKP (PI)_ = −6.462+3.759x−0.118x^2^					R^2^ = 0.868		*p*<0.05	
Y_AKP (MI)_ = 1.535+1.657x−0.052x^2^					R^2^ = 0.924		*p*<0.01	
Y_AKP (DI)_ = 0.964+0.351x−0.011x^2^					R^2^ = 0.883		*p*<0.05	

Values are means ± SD, n = 5. Mean values with the different superscripts in the same row are significantly different (*p*<0.05). AKP, alkaline phosphatase; γ-GT, γ-glutamyl transpeptidase; PI, proximal intestine; MI, mid intestine; DI, distal intestine.

#### Gene expression

**Figure 2:**
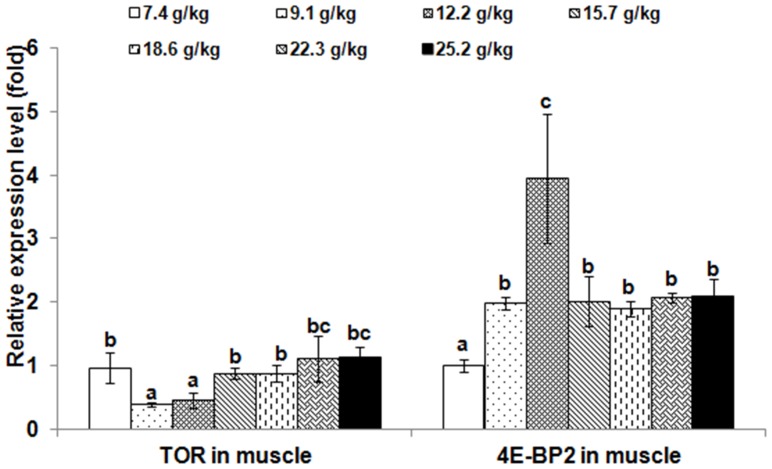
Effects of dietary Thr on gene expression in muscle of juvenile Jian carp. Values are means with standard deviations represented by vertical bars (n = 5). Different letter above bars indicated significant difference among treatments (*p*<0.05). Thr levels were 7.4, 9.1, 12.2, 15.7, 18.6, 22.3 and 25.2 g/kg diet respectively. *TOR*, *target of rapamycin*; *4E-BP2*, *eIF4E-binding protein2*.

**Figure 3:**
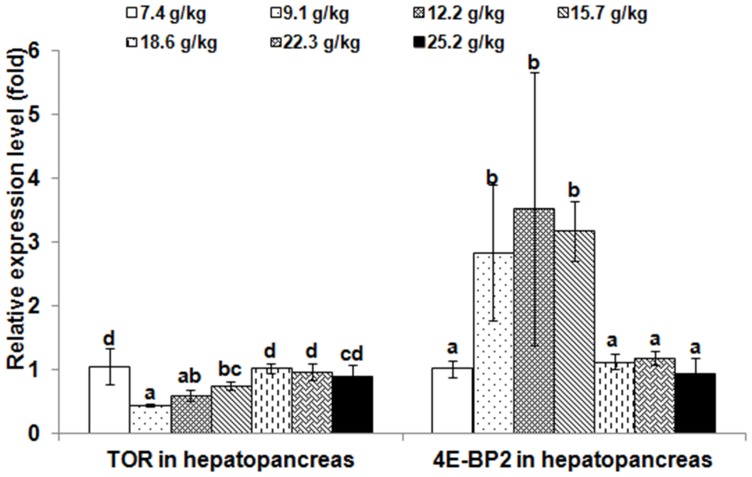
Effects of dietary Thr on gene expression in hepatopancreas of juvenile Jian carp. Values are means with standard deviations represented by vertical bars (n = 5). Different letter above bars indicated significant difference among treatments (*p*<0.05). Thr levels were 7.4, 9.1, 12.2, 15.7, 18.6, 22.3 and 25.2 g/kg diet respectively. *TOR*, *target of rapamycin*; *4E-BP2*, *eIF4E-binding protein2*.

**Figure 4:**
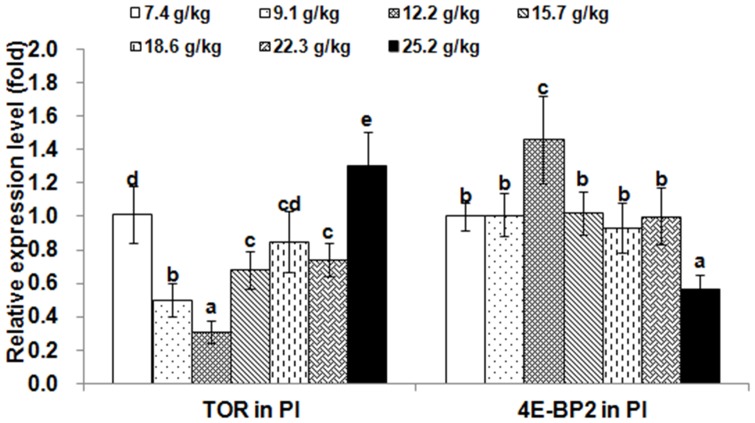
Effects of dietary Thr on gene expression in proximal intestine of juvenile Jian carp. Values are means with standard deviations represented by vertical bars (n = 5). Different letter above bars indicated significant difference among treatments (*p*<0.05). Thr levels were 7.4, 9.1, 12.2, 15.7, 18.6, 22.3 and 25.2 g/kg diet respectively. PI, proximal intestine; *TOR*, *target of rapamycin*; *4E-BP2*, *eIF4E-binding protein2*.

**Figure 5:**
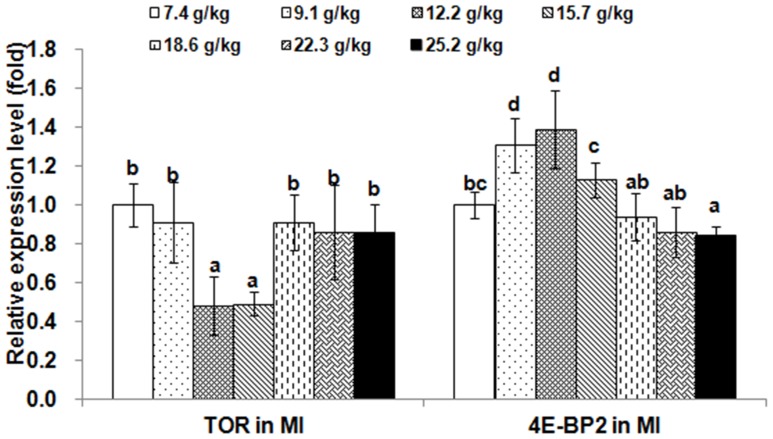
Effects of dietary Thr on gene expression in mid intestine of juvenile Jian carp. Values are means with standard deviations represented by vertical bars (n = 5). Different letter above bars indicated significant difference among treatments (*p*<0.05). Thr levels were 7.4, 9.1, 12.2, 15.7, 18.6, 22.3 and 25.2 g/kg diet respectively. MI, mid intestine; *TOR*, *target of rapamycin*; *4E-BP2*, *eIF4E-binding protein2*.

**Figure 6:**
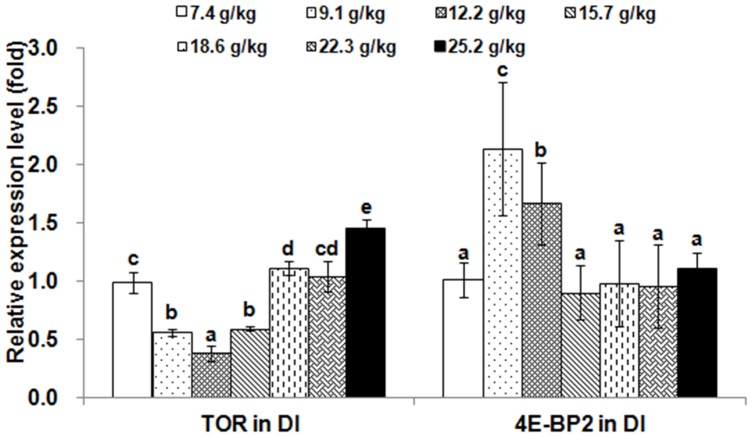
Effects of dietary Thr on gene expression in distal intestine of juvenile Jian carp. Values are means with standard deviations represented by vertical bars (n = 5). Different letter above bars indicated significant difference among treatments (*p*<0.05). Thr levels were 7.4, 9.1, 12.2, 15.7, 18.6, 22.3 and 25.2 g/kg diet respectively. DI, distal intestine; *TOR*, *target of rapamycin*; *4E-BP2*, *eIF4E-binding protein2*.

The relative expressions of *TOR* and 4E-BP2 genes in muscle and hepatopancreas are presented in Figure 2 and Figure 3, respectively. The *TOR* expression levels in muscle and hepatopancreas significantly decreased as dietary Thr increased to 9.1 g/kg diet (*p*<0.001), and then increased; whereas, the relative expressions of the *4E-BP2* gene in muscle and hepatopancreas were the highest in the group with Thr of 12.2 g/kg diet (*p*<0.001). The relative expressions of *TOR* and 4E-BP2 genes in PI, MI and DI are given in Figure 4, Figure 5 and Figure 6, respectively. The relative expressions of the *TOR* gene in PI, MI and DI significantly decreased in response to the increasing Thr levels from 7.4 to 12.2 g/kg diet, and increased thereafter (*p*<0.001). The relative expressions of the *4E-BP2* gene in PI and MI were significantly increased with increase of Thr levels up to 12.2 g/kg diet, and then decreased significantly (*p*<0.001). Meanwhile, the *4E-BP2* gene expression in DI significantly increased with Thr levels up to 9.1 g/kg diet (*p*<0.001), and significantly decreased with increased Thr levels up to 15.7 g/kg diet (*p*<0.001), then plateaued. The relative expressions of the *TOR* gene in PI and DI, and the *4E-BP2* gene in PI and MI responded quadractically to dietary Thr levels (Y*_TOR_*_ (PI)_ = 1.772−0.177x+0.006x^2^, R^2^ = 0.639, *p* = 0.130; Y*_TOR_*_ (DI)_ = 1.653−0.162x+0.006x^2^, R^2^ = 0.739, *p* = 0.068; Y*_4E-BP2_*_ (PI)_ = 0.399+0.116x−0.004x^2^, R^2^ = 0.627, *p* = 0.139; Y*_4E-BP2_*_ (MI)_ = 0.908+0.052x−0.002x^2^, R^2^ = 0.614, *p* = 0.149).

### *In vitro* analysis

#### Proliferation and differentiation of enterocytes

As given in Table 6, the MTT OD value was significantly higher in groups with Thr of 135, 170 and 205 mg/l than that in Thr-unsupplemented group (*p*<0.001). Cell protein content was significantly increased with increased levels of Thr (*p* = 0.027), and the lowest in Thr-unsupplemented group. The highest activities of AKP and Na^+^/K^+^-ATPase were observed in groups with 205 and 170 mg/l Thr respectively (AKP, *p* = 0.049; Na^+^/K^+^-ATPase, *p*<0.001). GOT and GPT activities were significantly enhanced as the Thr levels increased to 205 and 135 mg/l, respectively (*p*<0.001). The LDH activity and ammonia content in cell medium were significantly decreased with the increase of Thr levels up to 205 mg/l, and increased thereafter (LDH activity, *p*<0.001; ammonia content, *p* = 0.001) (Table 6). Regression analysis showed that cell protein content, Na^+^/K^+^-ATPase and LDH activities, and ammonia content were quadratic response to increased Thr levels (Table 6).

**Table 6:** 3-(4, 5-dimethylthiazol-2-yl)-2, 5-diphenyltetrazolium bromide optical density (MTT OD) value, protein content and enzymes activities of enterocytes, lactate dehydrogenase (LDH) activity and ammonia content in cell medium of enterocytes cultured with graded levels of Thr.

Thr level (mg/l)	0	135	170	205	240	275	*p*-value
Cell							
MTT OD value	0.165^ab^±0.012	0.197^c^±0.010	0.199^c^±0.010	0.211^c^±0.013	0.177^b^±0.016	0.150^a^±0.014	<0.001
Protein content (µg/well)	374.5^a^±8.0	381.9^ab^±10.2	383.1^ab^±17.1	396.6^bc^±21.3	395.3^bc^±2.4	405.1^c^±4.7	= 0.027
AKP (U/g protein)	1.91^a^±0.13	1.94^a^±0.15	1.99±^ab^±0.13	2.18^b^±0.12	2.12^ab^±0.14	2.13^ab^±0.12	= 0.049
Na^+^/K^+^-ATPase (U/g protein)	0.12^a^±0.02	0.28^c^±0.02	0.31^d^±0.03	0.29^cd^±0.02	0.24^b^±0.02	0.13^a^±0.01	<0.001
GOT (U/g protein)	1.57^a^±0.29	1.96^b^±0.32	2.23^bc^±0.00	3.38^d^±0.27	2.57^c^±0.27	2.24^bc^±0.26	<0.001
GPT (U/g protein)	2.34^a^±0.12	3.22^d^±0.12	2.68^c^±0.19	2.65^c^±0.22	2.60^bc^±0.00	2.42^ab^±0.13	<0.001
Cell medium							
LDH (U/g protein)	38.9^d^±4.8	25.8^c^±2.5	22.0^bc^±2.8	15.4^a^±2.4	17.8^ab^±2.4	20.8^b^±2.7	<0.001
Ammonia content (µmol/l)	1593^c^±126	1396^ab^±103	1367^ab^±92	1264^a^±39	1285^ab^±93	1432^ab^±73	= 0.001
Regressions							
Y_Cell protein content_ = 374+0.0007x+0.0004x^2^				R^2^ = 0.937		*p*<0.05	
Y_Na_^+^_/K_^+^_-ATPase_ = 0.117+0.003x−0.000009x^2^				R^2^ = 0.945		*p*<0.05	
Y_LDH_ = 39.4−0.163x+0.0003x^2^				R^2^ = 0.917		*p*<0.05	
Y_Ammonia content_ = 1604−2.84x+0.007x^2^				R^2^ = 0.803		*p* = 0.087	

Values are means ± SD, n = 4. Mean values with the different superscripts in the same row are significantly different (*p*<0.05). AKP, alkaline phosphatase; GOT, glutamate-oxaloacetate transaminase; GPT, glutamate-pyruvate transaminase.

#### Protein synthesis

The protein synthesis rate was 7.96±0.81 in enterocytes with 205 ml/l Thr, and 7.03±0.95 in enterocytes with no Thr (n = 8). Compared with the Thr-unsupplemented group, the protein synthesis rate was significantly increased by 13% in cells with 205 mg/l Thr (*p*<0.001).

## Discussion

### Thr improved fish growth

As an essential amino acid for fish, dietary Thr deficiency caused growth retardation of juvenile Jian carp in this study. The PWG, SGR, FI and FE were increased as dietary Thr increased to a certain point (Table 1), as reported before for Japanese flounder (*Paralichthys olivaceus*) [58] and Atlantic salmon (*Salmo salar*) [59]. Relative analysis showed that SGR was positively related to feed intake (r = +0.976, *p*<0.01) and feed efficiency (r = +0.883, *p*<0.01), suggesting that the improved growth by Thr was partly due to increased feed intake and feed utilization. Furthermore, body protein accretion makes an important contribution to fish weight gain [20]. The highest final body protein and lipid contents, and PRV in fish fed with 15.7 g Thr/kg diet further confirmed the best growth of fish in this group (Table 1). Similar pattern of body protein content was reported for Indian catfish (*Heteropneustes fossilis*) [30]. The beneficial effect of Thr on protein deposition may be ascribed to more efficient utilization of amino acids at this level of Thr [30]. GOT and GPT activities can be valuable indicators of the metabolic utilization of dietary amino acids by fish [60]. In teleosts, ammonia is the main end product of amino acid catabolism [61]. In our study, GOT and GPT activities in hepatopancreas and enterocytes were enhanced by Thr, while the ammonia contents in plasma and cell medium decreased. In channel catfish (*Ictalurus punctatus*), liver GOT activity was improved by a better dietary protein [62]. Ammonia production usually decreases with decreased amino acid catabolism rates [63]. Thus, the present data may partly suggest that Thr improved the utilization of amino acids. Furthermore, dietary Thr is mainly used for protein deposition which is associated with growth [22]. Based on PWG, the dietary Thr requirement of juvenile Jian carp was determined to be 16.25 g/kg diet or 51.3 g/kg protein by quadratic regression analysis, which is higher than that reported by Nose [9] for common carp, 39.0 g/kg protein. The higher growth rate of Jian carp than common carp may be a cause of the higher Thr requirement [64]. It's consistent with researches from our lab that Jian carp had higher lysine [23], inositol [31] and pyridoxine [32] requirements than common carp. Meanwhile, fish species may partly contribute to the difference of Thr requirement for various fish. As shown in Table 7, the Thr requirements for juvenile milkfish (*Chanos chanos* Forsskal) (45 g/kg protein) [65] and Indian major carp fingerling (*Cirrhinus mrigala* (Hamilton)) (46 g/kg protein) [66] were slightly higher than those for juvenile common carp (39 g/kg protein) [9], Indian catfish fingerling (34.2 g/kg protein) [30] and juvenile Japanese flounder (31.4 g/kg protein) [58], while the requirements for juvenile Atlantic salmon (23.5 g/kg protein) [59], juvenile hybrid striped bass (*Morone chrysops*♀×*M. saxatilis*♂) (26 g/kg protein) [67] and juvenile European sea bass (*Dicentrarchus labrax*) (23–26 g/kg protein) [68] were the lowest.

**Table 7:** Threonine requirements for different fishes.

Fish	g/kg protein	g/kg diet	Response Criteria	Reference
Juvenile common carp	39	15	Daily specific growth rate	Nose, 1979 [9]
Juvenile milkfish	45	18	WG	Borlongan and Coloso, 1993 [65]
Juvenile hybrid striped bass	26	9.7	WG	Keembiyehetty and Gatlin III, 1997 [67]
Juvenile European sea bass	23–26	11.2–12.6	WG	Tibaldi and Tulli, 1999 [68]
Juvenile Japanese flounder	31.4	15.7	WG	Alam et al., 2003 [58]
Indian major carp fingerling	46	18.4	WG	Ahmed et al., 2004 [66]
Indian catfish fingerling	34.2	13.7	WG	Ahmed, 2007 [30]
Juvenile Atlantic salmon	23.5	12.1	Thermal growth coefficient	Helland and Grisdale-Helland, 2011 [59]

### Thr enhanced digestive and absorptive capacity of fish

Fish growth is influenced by the digestive and absorptive capacity [1]. Digestive enzymes activities can directly reflect digestive ability [33]. Accordingly, we assayed the activities of digestive enzymes in hepatopancreas and intestine of juvenile Jian carp. The increased activities of trypsin, lipase and amylase in hepatopancreas and intestine in present study indicated that dietary Thr enhanced the digestive ability of fish (Table 4). Yet no other reports have shown the effect of Thr on digestive ability in fish. Digestive enzyme activity is known to be related to enzyme synthesis and secretion in fish [69]. The beneficial effect of Thr on activities of digestive enzymes may be partly related to the synthesis and secretion of enzymes. First, Thr may affect the synthesis of digestive enzymes. In terrestrial animal, Thr participated in amino acid composition of chymotrypsinogen, α-chymotrypsin and trypsinogen [70], and was necessary for amylase synthesis in pigeon pancreas [12]. Second, Thr may affect the secretion of digestive enzymes. In higher vertebrates, cholecystokinin (CCK) can regulate the release of exocrine pancreatic enzymes [1]. Konture et al. [71] reported that glycine, an important metabolite of Thr, stimulated the release of CCK in dog. Meanwhile, in terrestrial animal, digestive enzyme secretion can be regulated by many Ser/Thr kinases, and Thr residue phosphorylation is crucial for activating Ser/Thr kinases [72]. However, whether Thr improved the activities of digestive enzymes via regulating enzyme synthesis and secretion in fish needs further study. Moreover, fish exocrine pancreas is the main site for digestive enzyme synthesis and secretion [73]. The improved digestive enzymes activities may partly be due to improved hepatopancreas growth by Thr. In this study, hepatopancreas weight and protein content, and hepatosomatic index were enhanced with increasing Thr levels up to a certain point, suggesting that Thr improved the hepatopancreas growth of juvenile Jian carp. However, the mechanisms by which Thr enhanced hepatopancreas growth of fish remain to be elucidated.

Fish nutrient absorption is dependent on brush border enzyme activity [1] and intestinal folds height [7]. In this study, the activities of brush border enzymes, including AKP, Na^+^/K^+^-ATPase and γ-GT, and the folds height in all intestinal segments were increased by increased Thr levels up to a certain point, suggesting that Thr improved the absorptive ability of fish. To date, this is the first report about the relationship between Thr and absorptive function in fish. In piglets, Thr also increased midjejunum villus heights [15]. The absorptive function of fish is largely dependent on the growth and development of intestine [13]. In the current study, intestine weight and length, intestosomatic index, and relative gut length increased with increase of Thr levels up to a certain point, suggesting that Thr improved the intestinal growth and development of juvenile Jian carp. In terrestrial animal, a low Thr diet reduced the gut weight of rats [14] and the intestinal mass of piglets [74]. Intestinal development is related to protein content in rat [75]. Our present study showed that the protein contents in intestine and enterocytes were improved by Thr. Similarly, in rat, Thr increased the protein content in jejunum [14]. The important role of Thr in protein synthesis may be one of the primary causes of improved growth and development of intestine. Our *in vitro* study indicated that the enterocytes protein synthesis rate was enhanced by Thr, suggesting that Thr enhanced the protein synthesis ability of enterocytes in fish. However, this is the first report about the effect of Thr on enterocytes protein synthesis in fish. In rats, Thr increased the fractional synthesis rates of intestinal mucosal proteins in duodenum [14]. There may be two ways by which Thr affected protein synthesis ability. On one hand, Thr is an important material of protein synthesis. In piglets, the intestine extracts 60–80% of dietary Thr on the first pass and utilizes Thr for intestinal protein synthesis [16]. On the other hand, Thr may regulate protein synthesis ability via regulating the hormone level. Insulin plays an important role in regulation of protein synthesis [26]. Glycine, an important metabolite of Thr, increased insulin levels in pancreas of rat [76]. However, the mechanism whereby Thr improved the absorptive capacity of fish warrants further study.

The developmental growth and normal function of intestine depend on the proliferation and differentiation of enterocytes in piglets [77]. In order to further elucidate the mechanism of the effect of Thr on developmental growth and normal function of intestine, we investigated the effect of Thr on the proliferation and differentiation of Jian carp enterocytes *in vitro*. The proliferation of rat intestinal epithelial cells could be quantitatively measured by the MTT assay [78]. The AKP could reflect the differentiation of fish enterocytes [3], and Na^+^/K^+^-ATPase activity was associated with enterocytes function in rat [5]. Our present study showed that the MTT value, AKP and Na^+^/K^+^-ATPase activities in enterocytes were increased with Thr supplementation, indicating that Thr enhanced the proliferation and differentiation, and improved the function of Jian carp enterocytes. But there is no information concerning the effect of Thr on proliferation, differentiation and function of fish enterocytes. In pigs, Thr increased the goblet cells in duodenum and ileum [15]. Dahly et al. [79] reported that insulin-like growth factor I (IGF-I) increased enterocyte proliferation in rats. Thr participates in amino acid composition of IGF-I and plays an important role in maintaining the structure of IGF-I [80]. Meanwhile, integrin α5 plays a role in proliferation of enterocytes, and dietary Thr increased its gene expression in the ileum of piglets [81]. Many Ser/Thr kinases also play a pivotal role in regulating cell proliferation and differentiation in terrestrial animal [82]. Furthermore, the proliferation, differentiation and normal function of murine enterocytes rely on structural integrity of cells [83]. LDH release from cell is a marker for assessing integrity of epithelial cell [84]. In the present study, the LDH activity in cell medium was decreased by Thr, suggesting that Thr improved the integrity of enterocytes thereby enhanced the proliferation and differentiation of cells. Until now, no report has been conducted about the effect of Thr on structural integrity of enterocytes. The structural integrity of cells is partly related to their antioxidant ability. The improved structural integrity of enterocytes by Thr may be partially ascribed to the enhanced antioxidant ability of cells. Reduced glutathione (GSH) plays a critical role in cellular defenses against oxidative stress [85]. Glycine, an important metabolite of Thr, is an essential substrate of GSH synthesis [85]. Nevertheless, the mechanism of Thr improving proliferation and differentiation of fish enterocytes remains to be elucidated.

### Thr regulated *TOR* and *4E-BP*

As mentioned above, the protein contents in fish carcass, hepatopancreas, intestine and enterocytes, as well as the protein synthesis in enterocytes of Jian carp were improved by Thr. In mammals, the TOR signaling pathway plays an important role in regulating protein synthesis [25]. The 4E-BPs is one of the major downstream targets of TOR protein [26]. A recent study from our laboratory reported that intestinal protein synthesis of Jian carp was regulated by TOR molecule [29]. The present results showed that dietary Thr decreased the *TOR* gene expressions in muscle, hepatopancreas and intestine of Jian carp, while the *4E-BP2* gene expressions were up-regulated. To date, scanty data are reported about the relationship between Thr and the expressions of *TOR* and *4E-BP2* genes in fish. In terrestrial animal, only a few studies have reported the relationship between Thr and *4E-BP* gene, which showed that a low Thr diet decreased the *4E-BP1* gene expression in ileum of piglets [81]. These results showed that Thr may regulate the protein synthesis through the TOR pathway. In rainbow trout, the 4E-BP1 expression level in muscle was enhanced by a high dietary carbohydrate-to-protein ratio [86]. Additionally, dietary protein sources and branched chain amino acids levels regulated the *TOR* gene expression in liver of rainbow trout [87]. In zebrafish, L-leucine increased the phosphorylation levels of 4E-BP1 and activated the TOR signaling pathway under Diamond-Blackfan anemia [88]. These data confirmed our results that the nutritional status can regulate TOR signaling in fish as it does in mammals. However, the mechanisms by which dietary Thr regulates *TOR* and *4E-BP* gene expression are unclear. Gene expression is profoundly affected by the modulation of termination. In eukaryotes, Thr is one of the amino acid biosynthetic operons and can form hairpins involved in termination of gene expression [89]. Furthermore, RNA transcription is regulated by many proteins in eukaryotes [90]. As an essential amino acid, Thr participates in amino acid composition of many proteins involved in transcription regulation and plays an important role in regulating protein activity [91], [92], [93], [94]. In mouse, Thr residues participated in amino acid composition of Ets protein, which plays an important role in regulating transcription [91]. In transfected HeLa cells, Thr deficiency increased the activity of the *CHOP* promoter, which encodes a small nuclear protein that can influence gene expression [92]. Additionally, CCAAT-Enhancer Binding Protein-b (C/EBPb) is the essential master transcription factor in the liver and other major organs, and the phosphorylation of the Thr217 residue of mouse C/EBPb is essential for inducing the target gene [93]. Ser/Thr protein kinases can be activated through phosphorylation of Thr residues, and they play a crucial role in gene regulatory processes in mouse [94].

In conclusion, the present study indicated that Thr enhanced growth and increased capacities of digestive and absorptive which may be related to the improved growth and development of hepatopancreas and intestine. Thr also regulated *TOR* and *4E-BP2* gene expression in Jian carp. Furthermore, Thr improved proliferation and differentiation, as well as the protein synthesis of enterocytes. However, the mechanisms whereby Thr enhanced fish protein synthesis ability through regulating the expressions of *TOR* and *4E-BP2* genes warrant further study. The dietary Thr requirement of juvenile Jian carp was 16.25 g/kg diet or 51.3 g/kg protein based on the PWG.

## Supporting Information

Table S1 Composition, nutrients content and Thr content of the experimental diets. ^1^Essential amino acids (g/kg diet): arginine 12.4, histidine 3.9, isoleucine 8.99, leucine 14.4, lysine 14.7, methionine 6.23, phenylalanine 10.5, tryptophan 2.63, valine 9.78. Non-essential amino acids (g/kg diet): cystine 1.18, tyrosine 7.02, alanine 17.81, aspartic acid 31.06, glutamic acid 15.2. ^2^L-Thr was microencapsulated and contained 498.2 g Thr/kg. ^3^Per kilogram of mineral mixture (g/kg mixture): FeSO_4_·7H_2_O (197.0 g/kg Fe), 76.1 g; CuSO_4_·5H_2_0 (250.0 g/kg Cu), 1.20 g; ZnSO_4_·7H_2_O (225.0 g/kg Zn), 13.3 g; MnSO_4_·H_2_O (318.0 g/kg Mn), 4.09 g; KI (38.0 g/kg I), 2.90 g; NaSeO_3_ (10.0 g/kg Se), 2.50 g. All ingredients were diluted with CaCO_3_ to 1 kg. ^4^Per kilogram of vitamin mixture (g/kg mixture): retinyl acetate (172 mg/g), 0.800 g; cholecalciferol (12.5 mg/g), 0.480 g; DL-α-tocopherol acetate (500 g/kg), 20.000 g; menadione (500 g/kg), 0.200 g; thiamin nitrate (980 g/kg), 0.063 g; riboflavine (800 g/kg), 0.625 g; pyridoxine hydrochloride (980 g/kg), 0.755 g; cyanocobalamin (100 g/kg), 0.010 g; ascorbyl-2-polyphosphate(350 g/kg), 19.029 g; calcium-D-pantothenate (980 g/kg), 2.511 g; niacin (980 g/kg), 2.857 g; D-biotin (200 g/kg), 0.500 g; meso-inositol (980 g/kg), 52.857 g; folic acid (960 g/kg), 0.521 g. All ingredients were diluted with corn starch to 1 kg. ^5^Crude protein, crude fat and analyzed Thr were measured value. n-3 and n-6 contents calculated according to NRC [35] and Bell [88].
(DOC)

Table S2 Real-time primer sequences and thermocycling conditions for *target of rapamycin (TOR)*, *eIF4E-binding protein2 (4E-BP2)* and *β-actin* gene.
(DOCX)
