# Supplementary material for: Threonine Affects Intestinal Function, Protein Synthesis and Gene Expression of TOR in Jian Carp (Cyprinus carpio var. Jian)
Source: PLoS One. 2013 Jul 26;8(7):e69974. doi: 10.1371/journal.pone.0069974 (PMC3724917; doi:10.1371/journal.pone.0069974)
Supplement: Table S1 — Composition, nutrients content and Thr content of the experimental diets. 1Essential amino acids (g/kg diet): arginine 12.4, histidine 3.9, isoleucine 8.99, leucine 14.4, lysine 14.7, methionine 6.23, phenylalanine 10.5, tryptophan 2.63, valine 9.78. Non-essential amino acids (g/kg diet): cystine 1.18, tyrosine 7.02, alanine 17.81, aspartic acid 31.06, glutamic acid 15.2. 2L-Thr was microencapsulated and contained 498.2 g Thr/kg. 3Per kilogram of mineral mixture (g/kg mixture): FeSO4·7H2O (197.0 g/kg Fe), 76.1 g; CuSO4·5H20 (250.0 g/kg Cu), 1.20 g; ZnSO4·7H2O (225.0 g/kg Zn), 13.3 g; MnSO4·H2O (318.0 g/kg Mn), 4.09 g; KI (38.0 g/kg I), 2.90 g; NaSeO3 (10.0 g/kg Se), 2.50 g. All ingredients were diluted with CaCO3 to 1 kg. 4Per kilogram of vitamin mixture (g/kg mixture): retinyl acetate (172 mg/g), 0.800 g; cholecalciferol (12.5 mg/g), 0.480 g; DL-α-tocopherol acetate (500 g/kg), 20.000 g; menadione (500 g/kg), 0.200 g; thiamin nitrate (980 g/kg), 0.063 g; riboflavine (800 g/kg), 0.625 g; pyridoxine hydrochloride (980 g/kg), 0.755 g; cyanocobalamin (100 g/kg), 0.010 g; ascorbyl-2-polyphosphate(350 g/kg), 19.029 g; calcium-D-pantothenate (980 g/kg), 2.511 g; niacin (980 g/kg), 2.857 g; D-biotin (200 g/kg), 0.500 g; meso-inositol (980 g/kg), 52.857 g; folic acid (960 g/kg), 0.521 g. All ingredients were diluted with corn starch to 1 kg. 5Crude protein, crude fat and analyzed Thr were measured value. n-3 and n-6 contents calculated according to NRC [35] and Bell [88]. (DOC) [file pone.0069974.s001.doc]

**Table S1.**

Composition, nutrients content and Thr content of the experimental diets

| Ingredients | Diets (g/kg, dry diet) | | | | | | |
| --- | --- | --- | --- | --- | --- | --- | --- |
|  | Diet 1 | Diet 2 | Diet 3 | Diet 4 | Diet 5 | Diet 6 | Diet 7 |
| Casein | 175.2 | 175.2 | 175.2 | 175.2 | 175.2 | 175.2 | 175.2 |
| Gelatin | 43.8 | 43.8 | 43.8 | 43.8 | 43.8 | 43.8 | 43.8 |
| Amino acid mix^1^ | 155.8 | 155.8 | 155.8 | 155.8 | 155.8 | 155.8 | 155.8 |
| L-Thr^2^ | 0.0 | 5.0 | 11.0 | 17.1 | 23.1 | 29.1 | 35.1 |
| Ala (99%) | 4.4 | 3.8 | 3.0 | 2.3 | 1.5 | 0.8 | 0 |
| Asp (99%) | 6.6 | 5.7 | 4.5 | 3.4 | 2.3 | 1.1 | 0 |
| Glu (98.5%) | 7.3 | 6.3 | 5.0 | 3.8 | 2.5 | 1.3 | 0 |
| Corn starch | 280.0 | 277.5 | 274.8 | 271.7 | 268.9 | 266.0 | 263.2 |
| α-starch | 205.9 | 205.9 | 205.9 | 205.9 | 205.9 | 205.9 | 205.9 |
| Fish oil | 28.0 | 28.0 | 28.0 | 28.0 | 28.0 | 28.0 | 28.0 |
| Soya bean oil | 18.9 | 18.9 | 18.9 | 18.9 | 18.9 | 18.9 | 18.9 |
| Cellulose | 30.0 | 30.0 | 30.0 | 30.0 | 30.0 | 30.0 | 30.0 |
| Mineral mix^3^ | 10.0 | 10.0 | 10.0 | 10.0 | 10.0 | 10.0 | 10.0 |
| Vitamin mix^4^ | 10.0 | 10.0 | 10.0 | 10.0 | 10.0 | 10.0 | 10.0 |
| Ca(H_2_PO_4_)_2_ | 22.3 | 22.3 | 22.3 | 22.3 | 22.3 | 22.3 | 22.3 |
| Choline chloride | 1.30 | 1.30 | 1.30 | 1.30 | 1.30 | 1.30 | 1.30 |
| Ethoxyquin | 0.500 | 0.500 | 0.500 | 0.500 | 0.500 | 0.500 | 0.500 |
| Nutrient content (g/kg, dry diet)^5^ | | | | | | | |
| Crude protein | 316.7 | 317.0 | 316.9 | 316.0 | 316.4 | 316.8 | 317.2 |
| Crude fat | 49.0 | 48.7 | 48.9 | 49.1 | 49.0 | 49.2 | 48.8 |
| Analyzed threonine | 7.4 | 9.1 | 12.2 | 15.7 | 18.6 | 22.3 | 25.2 |
| n-3 | 10.0 | 10.0 | 10.0 | 10.0 | 10.0 | 10.0 | 10.0 |
| n-6 | 10.0 | 10.0 | 10.0 | 10.0 | 10.0 | 10.0 | 10.0 |

^1^Essential amino acids (g/kg diet): arginine 12.4, histidine 3.9, isoleucine 8.99, leucine 14.4, lysine 14.7, methionine 6.23, phenylalanine 10.5, tryptophan 2.63, valine 9.78. Non-essential amino acids (g/kg diet): cystine 1.18, tyrosine 7.02, alanine 17.81, aspartic acid 31.06, glutamic acid 15.2.

^2^L-Thr was microencapsulated and contained 498.2 g Thr/kg.

^3^Per kilogram of mineral mixture (g/kg mixture): FeSO_4_·7H_2_O (197.0 g/kg Fe), 76.1 g; CuSO_4_·5H_2_0 (250.0 g/kg Cu), 1.20 g; ZnSO_4_·7H_2_O (225.0 g/kg Zn), 13.3 g; MnSO_4_·H_2_O (318.0 g/kg Mn), 4.09 g; KI (38.0 g/kg I), 2.90 g; NaSeO_3_ (10.0 g/kg Se), 2.50 g. All ingredients were diluted with CaCO_3_ to 1 kg.

^4^Per kilogram of vitamin mixture (g/kg mixture): retinyl acetate (172 mg/g), 0.800 g; cholecalciferol (12.5 mg/g), 0.480 g; DL-α-tocopherol acetate (500 g/kg), 20.000 g; menadione (500 g/kg), 0.200 g; thiamin nitrate (980 g/kg), 0.063 g; riboflavine (800 g/kg), 0.625 g; pyridoxine hydrochloride (980 g/kg), 0.755 g; cyanocobalamin (100 g/kg), 0.010 g; ascorbyl-2-polyphosphate(350 g/kg), 19.029 g; calcium-D-pantothenate (980 g/kg), 2.511 g; niacin (980 g/kg), 2.857 g; D-biotin (200 g/kg), 0.500 g; meso-inositol (980 g/kg), 52.857 g; folic acid (960 g/kg), 0.521 g. All ingredients were diluted with corn starch to 1 kg.

^5^Crude protein, crude fat and analyzed Thr were measured value. n-3 and n-6 contents calculated according to NRC [35] and Bell [88].
