# Supplementary material for: Threonine Affects Intestinal Function, Protein Synthesis and Gene Expression of TOR in Jian Carp (Cyprinus carpio var. Jian)
Source: PLoS One. 2013 Jul 26;8(7):e69974. doi: 10.1371/journal.pone.0069974 (PMC3724917; doi:10.1371/journal.pone.0069974)
Supplement: Table S2 — Real-time primer sequences and thermocycling conditions for target of rapamycin (TOR), eIF4E-binding protein2 (4E-BP2) and β-actin gene. (DOCX) [file pone.0069974.s002.docx]

**Table S2.**

Real-time primer sequences and thermocycling conditions for *target of rapamycin (TOR)*, *eIF4E-binding protein2 (4E-BP2)* and *β-actin* gene

| Gene | Sequences of Primers | Thermocycling conditions | GenBank accession no. |
| --- | --- | --- | --- |
| *TOR* | | | |
| forward | 5’-ATC ATA CGC ATC CAG TCC ATT G-3’ | 40 cycles of 95^o^C 10sec, 95^o^C 5sec, 60^o^C 53 sec | FJ899680 |
| reverse | 5’-GGT CAT TAG CCA GTA GAG TGT TC-3’ |  |  |
| *4E-BP2* | | | |
| forward | 5’-GCT ACC TCA CGA CTA TTG C-3’ | 40 cycles of 95^o^C 10sec, 95^o^C 5sec, 59.5^o^C 30 sec | HQ010440 |
| reverse | 5’-TTC TTG CTT GTC ACT CCT G-3’ |  |  |
| *β-actin* | | | |
| forward | 5’-CGT GAT GGA CTC TGG TGA TG-3’ | 40 cycles of 95^o^C 10sec, 95^o^C 5sec, 60^o^C 30 sec | M24113 |
| reverse | 5’-TCG GCT GTG GTG GTG AAG-3’ |  |  |
